# Live mechanistic assessment of localized cardiac pumping in mammalian tubular embryonic heart

**DOI:** 10.1117/1.JBO.25.8.086001

**Published:** 2020-08-05

**Authors:** Shang Wang, Irina V. Larina

**Affiliations:** a Stevens Institute of Technology, Department of Biomedical Engineering, Hoboken, New Jersey, USA; b Baylor College of Medicine, Department of Molecular Physiology and Biophysics, Houston, Texas, USA

**Keywords:** optical coherence tomography, embryonic heart, Doppler, hemodynamics, cardiac pumping, mouse

## Abstract

**Significance:** Understanding how the valveless embryonic heart pumps blood is essential to elucidate biomechanical cues regulating cardiogenesis, which is important for the advancement of congenital heart defects research. However, methods capable of embryonic cardiac pumping analysis remain limited, and assessing this highly dynamic process in mammalian embryos is challenging. New approaches are critically needed to address this hurdle.

**Aim:** We report an imaging-based approach for functional assessment of localized pumping dynamics in the early tubular embryonic mouse heart.

**Approach:** Four-dimensional optical coherence tomography was used to obtain structural and Doppler hemodynamic imaging of the beating heart in live mouse embryos at embryonic day 9.25. The pumping assessment was performed based on the volumetric blood flow rate, flow resistance within the heart tube, and pressure gradient induced by heart wall movements. The relation between the blood flow, the pressure gradient, and the resistance to flow were evaluated through temporal analyses and Granger causality test.

**Results:** In the ventricles, our method revealed connections between the temporal profiles of pressure gradient and volumetric blood flow rate. Statistically significant causal relation from the pressure gradient to the blood flow was demonstrated. Our analysis also suggests that cardiac pumping in the early ventricles is a combination of suction and pushing. In contrast, in the outflow tract, where the conduction wave is slower than the blood flow, we did not find significant causal relation from pressure to flow, suggesting that, different from ventricular regions, the local active contraction of the outflow tract is unlikely to drive the flow in that region.

**Conclusions:** We present an imaging-based approach that enables localized assessment of pumping dynamics in the mouse tubular embryonic heart. This method creates a new opportunity for functional analysis of the pumping mechanism underlying the developing mammalian heart at early stages and could be useful for studying biomechanical changes in mutant embryonic hearts that model congenital heart defects.

## 1 Introduction

How a valveless embryonic heart tube pumps blood has been a long-standing question.^1^ Early observations led to the traditional view that the tubular heart functions with peristaltic waves pushing the blood through the heart for circulation.^2^^,^^3^ However, hemodynamic data in recent decades described features of blood flow that could not be explained by the classic peristaltic pumping model.^4^^,^^5^ Notably, one study on zebrafish embryos showed that the valveless heart does not drive blood circulations through peristalsis, but rather through a suction mechanism.^6^ Such suction-like pumping was also observed in chick embryos.^7^ The complexity of the tubular heart pumping dynamics was emphasized in a review by Männer et al.^1^ about a decade ago, indicating that further functional analyses are needed to elucidate how the early embryonic heart works. So far, studies have mainly focused on avian and teleost models,^4^^–^^7^ leaving the cardiac pumping mechanism in mammalian embryos largely unexplored.

Live embryonic cardiodynamic imaging is required for mechanistic analysis. However, the relatively small size and fast dynamics of the heart at this early developmental stage demand a high spatiotemporal resolution, leaving limited methods available for assessment of the pumping process. High-frequency ultrasound has been used to capture the cardiac hemodynamics and wall movements,^7^^–^^9^ but the lack of 3D visualization and the inability to resolve structural details prevent its use for systematic and accurate pumping characterization. High-resolution, high-speed optical modalities have enabled promising approaches. By employing postacquisition synchronization,^10^ 4D (3D + time) cardiodynamic imaging in zebrafish embryos was achieved with confocal microscopy,^11^ and simultaneous analyses of heart wall motions and movements of individual blood cells produced exciting insights into the pumping mechanism.^6^ Recently, advancements in light-sheet microscopy allowed for direct ultrafast volumetric imaging of the beating heart in zebrafish embryos at the single-cell level in real time,^12^ making it more efficient and convenient to conduct biomechanical analysis. However, with the current imaging depths achieved by these two modalities, capturing the entire heart tube in mammalian embryos, such as the mouse, is challenging.

Optical coherence tomography (OCT) is a 3D imaging modality^13^ providing unique imaging scales and contrasts that are increasingly utilized for developmental biology studies^14^ and particularly for investigations of the embryonic heart.^15^^–^^18^ By employing near-infrared light and low-coherence interferometry, OCT enables a millimeter-level imaging depth in scattering tissues with a microscale resolution, ideal for covering the entire mouse heart in cultured embryos at midgestation stages and resolving fine cardiac structures, such as the endocardium.^16^ In addition to structural imaging, OCT allows for the use of dynamic contrast for functional imaging of blood flow,^19^ where quantitative imaging of cardiodynamics and hemodynamics of the mouse embryonic heart can be achieved simultaneously with Doppler OCT.^20^ The high imaging speed of OCT enables a sufficient temporal resolvability to capture heart movements in mouse embryos, either through direct volumetric data acquisition with an ultrafast system^21^ or through postacquisition synchronization.^16^ Using sequential acquisition, retrospective gating, and synchronization, we routinely achieve a 100-Hz volume rate in reconstruction for combined 4D structural and hemodynamic imaging of the mouse embryonic heart.^20^ This represents a sampling rate of over ∼50 times of the heart rate at the studied developmental stages, providing valuable information for time-resolved mechanistic investigations. Biomechanical factors, such as the flow-induced shear force, are essential in driving and regulating cardiogenesis;^22^^,^^23^ since the assessment of pumping investigates the fundamental process of flow generation, pumping analysis is of critical value for understanding the early cardiac development and congenital heart defects. Although OCT has been employed for biomechanical analysis of embryonic hearts,^24^^–^^26^ to our knowledge, no methods are currently available for assessment of the pumping process.

To address this need, we report an OCT-based approach for functional assessment of pumping dynamics in the mouse tubular embryonic heart. Inspired by the recent biomechanical model,^27^ the method investigates the effects of the pressure gradient induced by heart wall movements and the viscous resistance on the volumetric blood flow through high-resolution temporal analysis and statistical causality test. Although structurally simpler than the mature heart, the early embryonic heart tube presents complex dynamic activities.^28^^–^^30^ In particular, the conduction wave velocity differs among regions of the tubular heart,^31^^–^^33^ as explained by their different developmental cell origins.^34^ Particularly in the mouse model, it has been shown that over early development before embryonic day 9.5 (E9.5), the conduction velocity, responsible for active regional contraction, increases only in the ventricles, leaving the conduction velocity significantly lower in the atria, atrioventricular ring, interventricular groove, and outflow tract.^35^ This motivated us to focus on localized pumping assessment on cardiac regions with relatively homogeneous electrophysiological characteristics. With this approach, we performed analyses on the ventricles and the outflow tract of E9.25 mouse embryonic hearts. The temporal analyses presented detailed pumping processes in the ventricles that suggest combined pushing and suction activities. In contrast to ventricles, the assessment revealed distinct pumping dynamics from the outflow tract where blood flow stays ahead of active local contraction waves. Our results indicate that the presented OCT-based functional approach can be a useful tool to characterize cardiac pumping in the tubular embryonic heart, which could bring new opportunities to study biomechanics in normal and defected cardiogenesis.

## 2 Materials and Methods

### 2.1 Mouse Embryo Manipulations

**Fig. 1:**
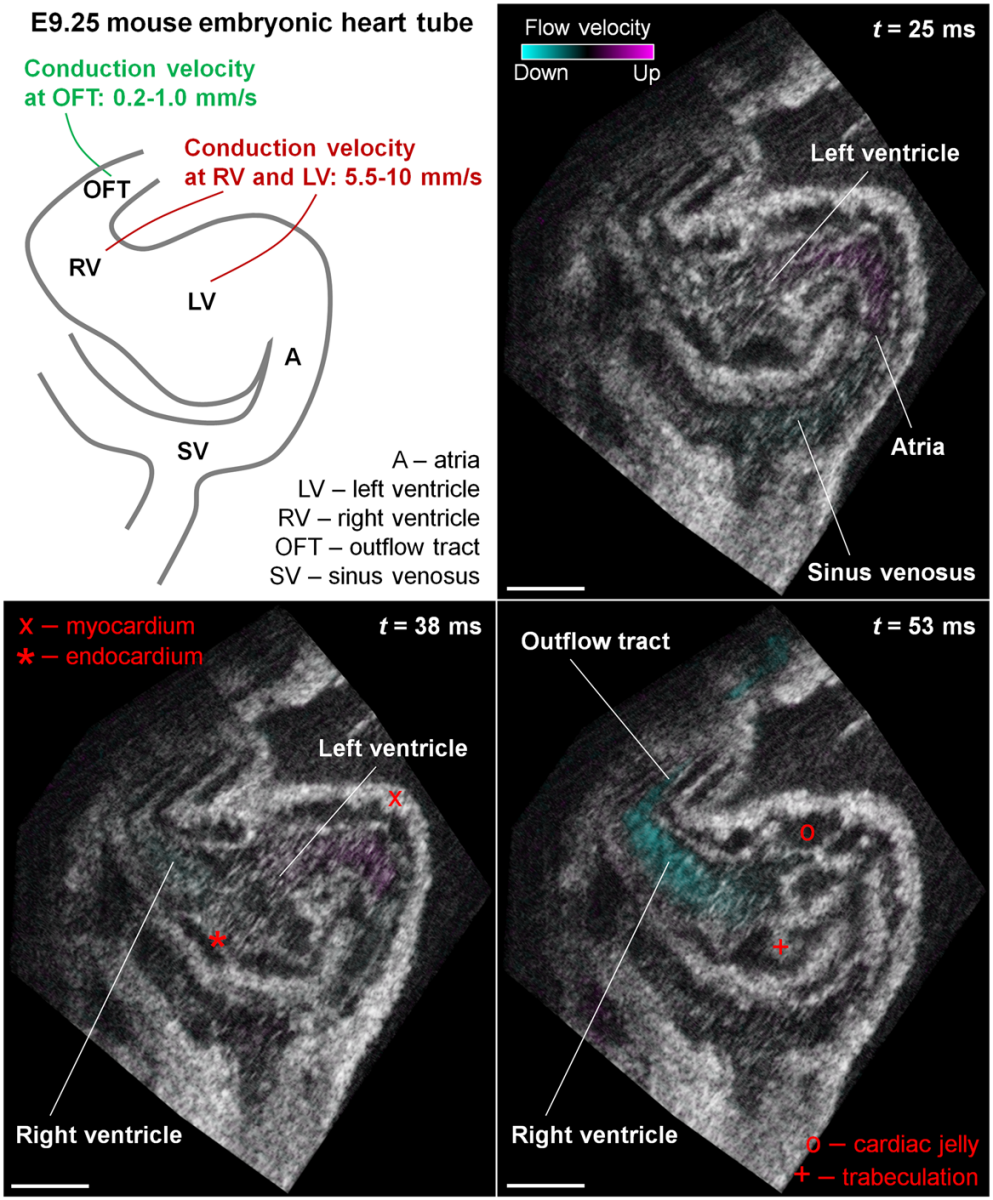
Structural and Doppler 4D-OCT imaging of E9.25 embryonic heart (Video 1). Illustration and representative frames through the 4D-OCT reconstruction at different phases of cardiac cycle showing the relative locations of sinus venosus, atria, left ventricle, right ventricle, and outflow tract. The OCT images are *en face* visualizations. Time stamps correspond to the time in Video 1. The approximate conduction velocity values at this stage are adopted from previous work.^35^ Scale bars are 100  μm (Video 1, mov, 9.39 MB [URL: https://doi.org/10.1117/1.JBO.25.8.086001.1]).

Wild-type CD-1 mice were used in this study. All animal manipulations have been approved by the Institutional Animal Care and Use Committee at Baylor College of Medicine, and experiments followed the approved procedures and guidelines. Embryos were dissected live at E9.25 with the intact yolk sac in 37°C culture medium following established protocols.^36^ After recovery in the incubator at 37°C and 5% ${\mathrm{CO}}_{2}$, embryos were positioned with the heart facing up and placed on the OCT imaging stage maintained at 37°C and 5% ${\mathrm{CO}}_{2}$ within an incubator.^37^ At E9.25, the interventricular groove separates the left and right ventricles,^35^^,^^38^ as illustrated in Fig. 1. This morphological feature of the heart tube allows for distinguishing the two ventricles. The culture environment has been previously optimized for early stage mouse embryonic development.^37^ A normal heart rate (∼1.5 to 2 Hz) and an active blood cell circulation were monitored in real time with OCT during imaging. Three stage-matched embryos were used for imaging and analysis of cardiac pumping in the right ventricle, left ventricle, and outflow tract; one embryo per each of the three cardiac regions. Different regions were studied in different embryos to ensure the best OCT visualization of the cardiac region under study and strong axial component of blood flow for Doppler analysis.

### 2.2 OCT System and 4D Imaging

We employed a customer-built spectral-domain OCT system^37^ with an ∼810-nm central wavelength and an ∼110-nm bandwidth. The system axial resolution was measured as ∼5  μm in tissue (assumed refractive index of 1.4), and the transverse resolution was measured as ∼4  μm. The B-scan rate was set at 100 Hz with an A-scan rate of ∼68  kHz. The sample arm was placed in a 37°C, 5% ${\mathrm{CO}}_{2}$ incubator for embryo experiments. Toward 4D reconstruction of the beating heart, the data were acquired as a single, dense 3D set covering the embryonic heart.^19^ The frame sequence was split into individual heartbeat cycles, then each subsequence was assigned to a spatial location separated from neighboring subsequences by a small step. The subsequences for all spatial locations have been synchronized to the same phase of the heartbeat cycles to reveal a 4D cardiodynamic dataset. The postprocessing method for a combined 4D structural and Doppler OCT reconstruction of the entire beating embryonic heart was previously established.^20^ Briefly, based on the OCT complex signal I=x+iy, structural images were obtained with the intensity |I| of each pixel in the logarithm scale, and Doppler images were achieved by the windowed Kasai autocorrelation function,^39^ where axial flow velocity $v_{a}$ was mapped to each pixel. The calculation for the spatial location (m,n) was via

$$v_{a}=\frac{\lambda f_{A}}{4\pi n}\;\arctan\;\frac{\overset{M}{\underset{m=1}{\sum }}\overset{N-1}{\underset{n=1}{\sum }}(x_{m,n+1}y_{m,n}-y_{m,n+1}x_{m,n})}{\overset{M}{\underset{m=1}{\sum }}\overset{N-1}{\underset{n=1}{\sum }}(x_{m,n+1}x_{m,n}+y_{m,n+1}y_{m,n})},$$ (1)

where λ is the central wavelength of light, $f_{A}$ is the A-scan rate of the OCT system, n is the refractive index of blood assumed as 1.4,^20^ and M×N is the spatial window in pixels. The synchronization was conducted based on structural images, which guided the rearrangement of corresponding Doppler images for registered 4D cardiodynamics and hemodynamics of the whole heart. Volumetric rendering and visualizations were performed in the Imaris software (Bitplane). An example for combined 4D structural and Doppler imaging of the entire E9.25 mouse embryonic heart is shown in Fig. 1 and Video 1, where the myocardium, endocardium, cardiac jelly, and trabeculation can be well distinguished and the color-coded blood flow is clearly seen. After the 4D reconstruction of the entire beating heart, specific cardiac regions were selected for pumping analysis. The pumping assessment was performed in the right ventricle, left ventricle, and outflow tract of the heart tube. According to previously published studies,^35^ the ventricles and the outflow tract have distinct electrophysiological properties with different conduction velocities, as illustrated in Fig. 1.

### 2.3 Biomechanical Model and Measurements

Based on the recent work^27^ from Bulk et al., by modeling the heart tube as a differential cylindrical volume, neglecting radius variations along the cross section of tube, and assuming axisymmetric wall movements, the final pressure gradient ΔP over the distance z along the tube is contributed by two factors, the pressure gradient $\Delta P_{\text{wall}}$ induced by heart wall movements and the viscous resistance R, with $\Delta P=\Delta P_{\text{wall}}-R$. The heart-wall-movement-induced pressure gradient $\Delta P_{\text{wall}}$ is determined by the luminal area changing rate normalized by the area, described as

$$\Delta P_{\mathrm{wall}}=\Delta (-\frac{\mu }{A}\frac{dA}{dt}),$$ (2)

where A is the luminal area, μ is the viscosity, and t is the time. The viscous resistance R is also related to the luminal area though

$$R=\frac{\pi }{4}\frac{\mu Cu}{A}\Delta z,$$ (3)

where u is the flow velocity and C is a constant of velocity profile. Thus, a smaller luminal area creates a dominantly high resistance to flow, whereas a larger area leaves a minimal flow resistance. In our method, both $\Delta P_{\text{wall}}$ and R were taken into account for analyzing the pumping process. However, due to the difficulty of accurately measuring certain parameters, including the blood viscosity and the flow profile, the final overall pressure gradient ΔP was not quantified. Instead, we separately assessed the pressure gradient induced by heart wall movements and the averaged viscous flow resistance and evaluated their combined effects on the volumetric blood flow. Specifically, the blood viscosity was assumed as constant over the localized region of assessment,^40^ and $\Delta P_{\text{wall}}$ was thus characterized as

$$\Delta P_{\mathrm{wall}}\propto \Delta (-\frac{1}{A}\frac{dA}{dt}).$$ (4)

Also similar to the previous study,^27^ the flow resistance was evaluated as

$$R\propto \frac{1}{A_{a}},$$ (5)

with a normalization to the range of [0, 1], where $A_{a}$ is the averaged area of endocardial lumen in the region. Such strategies simplify the measurements of essential parameters and improve the efficiency of this method for pumping analysis.

**Fig. 2:**
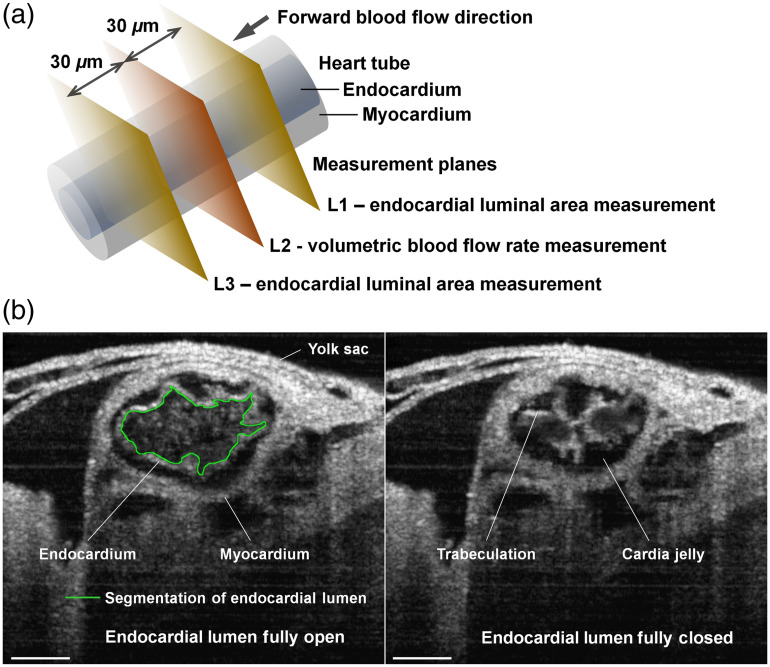
(a) Illustration showing locations L1, L2, and L3 along the heart tube in the direction of forward flow to measure the dynamics of endocardial lumen at L1 and L3 and blood flow at L2. All measurement planes are set perpendicular to the tube axis. (b) Structural 4D-OCT imaging of E9.25 embryonic heart showing the open and closed endocardial lumen. An example of lumen segmentation is presented with the green line for the fully open endocardial lumen. Scale bars are 100  μm.

Each localized pumping assessment was conducted with three measurement planes perpendicular to the heart tube axis within the 4D cardiodynamic dataset at the selected region of interest, as illustrated in Fig. 2. The planes were spaced by ∼30  μm and named L1, L2, and L3 in the direction of forward blood flow. All measurements were performed at the intersections between the planes and the endocardial lumen. The endocardial layer outlining the lumen can be well delineated from the cross sections in the OCT images, as seen in Fig. 2. The measurements were conducted for all time points over one heartbeat cycle. At L1 and L3, the endocardial lumen area A was measured with three manual segmentations in ImageJ (an example shown in Fig. 2), and the data were plotted as mean±std. The mean values were used to assess the pressure gradient induced by heart wall movements between L1 and L3 with Eq. (4), and also to calculate the averaged area $A_{a}$ for evaluating the resistance to flow with Eq. (5). At L2, the volumetric flow rate V was quantified as the integration of absolute blood flow velocity over the endocardial lumen area through

$$V=\int \frac{v_{a}}{\cos\;\theta }dA,$$ (6)

where θ is the angle between the blood flow direction and the imaging beam. The measured parameters were plotted over time for detailed temporal analyses. Granger causality test^41^ was employed for statistical evaluation of the causal relation between dynamics, specifically to determine whether the observed blood flow is caused by the localized pressure gradient produced by heart wall movements.

## 3 Results

**Fig. 3:**
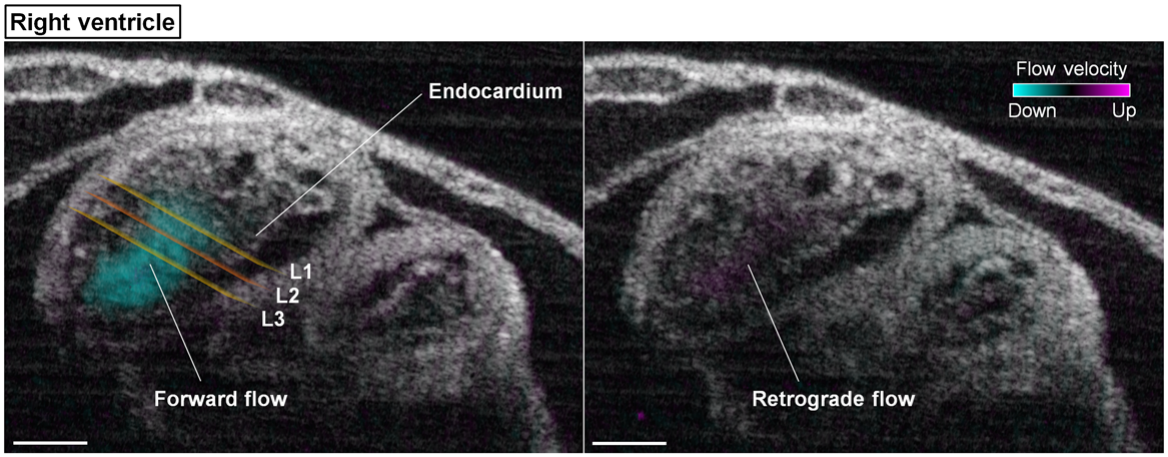
Structural and Doppler 4D-OCT imaging of the right ventricle of E9.25 embryonic heart (Video 2). The positions of the measurement planes L1, L2, and L3 are shown in the left panel. Scale bars are 100  μm (Video 2, mov, 8.59 MB [URL: https://doi.org/10.1117/1.JBO.25.8.086001.2]).

In the right ventricle of E9.25 embryonic heart, both forward and retrograde flows were observed, as shown in Fig. 3 and Video 2. Over the heartbeat cycle, the retrograde flow formed at the beginning of the lumen opening, which switched to forward flows during the process of luminal opening. The forward flows were then maintained as the right ventricle continued to relax and reached the most expanded state, as well as over the entire contraction phase.

**Fig. 4:**
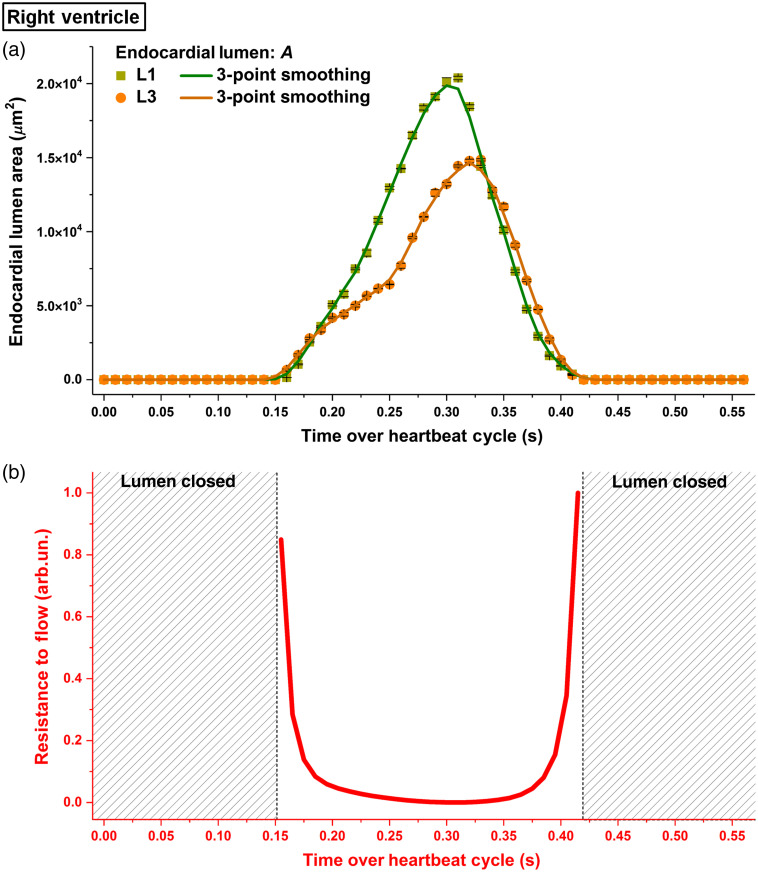
(a) Temporal profiles of endocardial lumen area at L1 and L3 at the right ventricle of E9.25 embryonic heart and (b) the corresponding normalized resistance to flow between L1 and L3. The luminal area in (a) is plotted as mean±std. Data are from one embryo.

From the plot of endocardial lumen area over time shown in Fig. 4, the lumen at L1 and L3 opened and closed at approximately the same time, but the time of the maximal luminal area at L3 was delayed relative to L1. Between L1 and L3, the dynamic processes revealed distinct profiles with L3 having a smaller luminal area at the most relaxed state and a generally slower area changing rate, which was especially obvious at the beginning of the right ventricle relaxation. The flow resistance profile as a result of the luminal area change presented a high resistance to flow when the lumens were just opening and were about to close, as shown in Fig. 4.

**Fig. 5:**
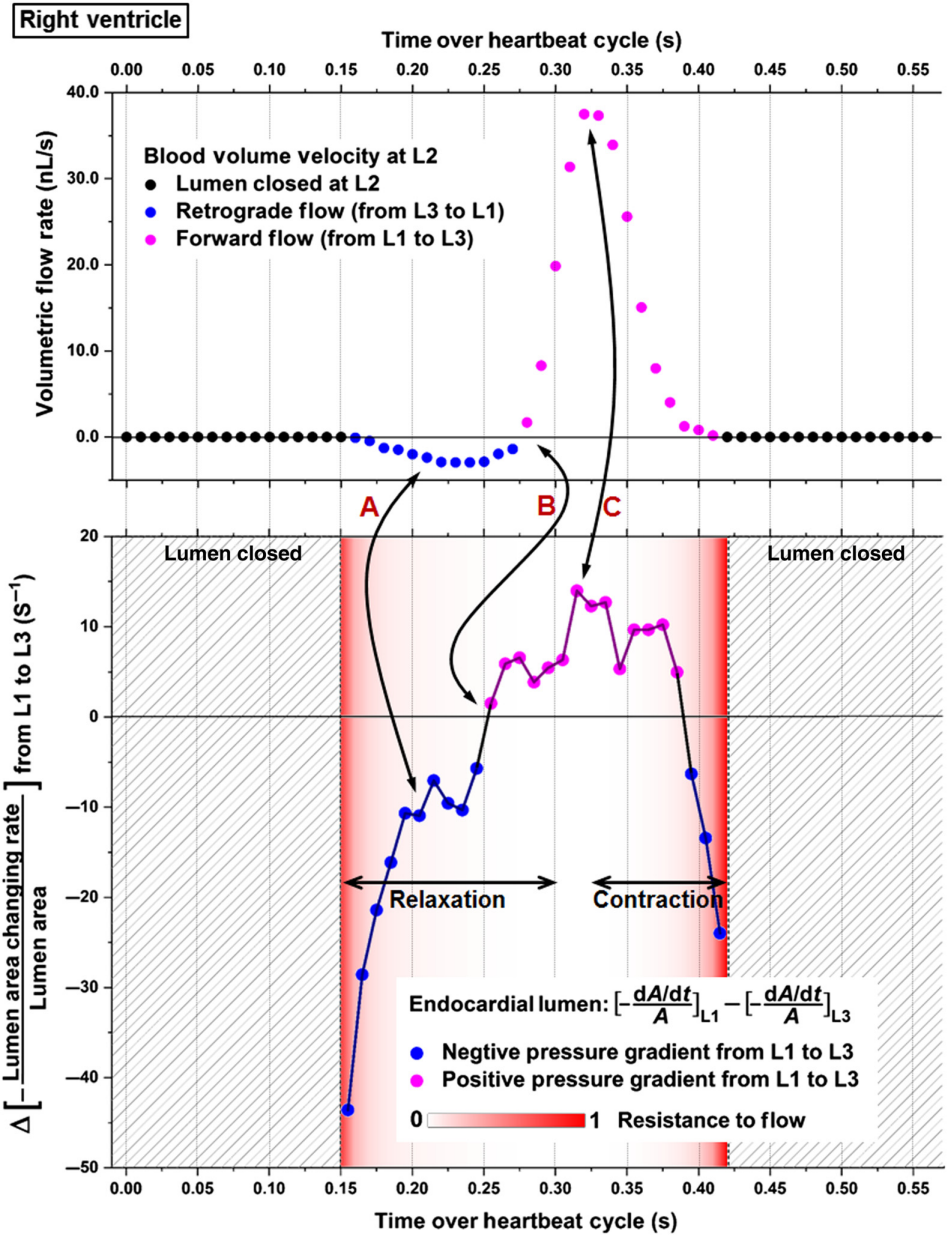
The relation between pressure gradient, resistance to flow, and volumetric flow rate at the right ventricle of E9.25 embryonic heart. Data are from one embryo.

The calculated pressure gradient produced by heart wall movements and the volumetric flow rate at L2 are shown in Fig. 5. The flow resistance (color coded in red) was overlaid with the pressure gradient profile for demonstration of their contribution to flow dynamics. It can be seen that at the beginning of the luminal relaxation, a negative pressure gradient from L1 to L3 formed and lasted for ∼100  ms, which coincided with the timing of the retrograde flow (double arrow A). As the luminal opening proceeded, the pressure gradient from L1 to L3 changed to positive, after which the flow switched its direction to forward (double arrow B). As the positive pressure gradient reached its peak, the forward flow rate also reached its maximum, though with a small time delay (double arrow C). These observations suggest coordination between the pressure gradient induced by heart wall movements and the volumetric blood flow. The resistance to flow, however, exhibited less pronounced effect on the flow dynamics than the heart-wall-movement-induced pressure gradient. In particular, at the beginning of luminal opening, as the flow resistance is high, despite a very strong negative pressure gradient from L1 to L3, the retrograde flow did not show an instantly large magnitude but rather gradually increased. In addition, as the lumen was closing, the negative pressure gradient from L1 to L3 failed to induce a retrograde flow due to high resistance to flow at the same time. During the heartbeat phase of relatively low flow resistance (below 0.1), we tested the causal relation between the pressure gradient and the volumetric blood flow using Granger causality test at different lag values. The lag represents the temporal data point, with the lag values of 1, 2, and 3 corresponding to the time differences of 10, 20, and 30 ms, respectively. As shown in Table 1, the only statistical significance was detected at the lag of two temporal data points in the causal direction of pressure gradient to flow, indicating that, in the right ventricle, the localized pressure gradient produced the specific flows while the viscous resistance is low.

**Table 1:** Granger causality test for the right ventricle of E9.25 embryonic heart. **p≤0.01.

Causal direction	Lag=1	Lag=2	Lag=3
Pressure gradient → flow	p=0.859	p=0.006**	p=0.149
Flow → pressure gradient	p=0.346	p=0.544	p=0.768

Based on the luminal area data shown in Fig. 4, we marked the time periods for common contractions and relaxations of this localized right ventricle region in Fig. 5. Interestingly, it can be seen that the heart wall relaxation generated both negative and positive pressure gradients that led to the corresponding retrograde and forward flows, suggesting suction process, while over the contraction, the heart wall movements created a positive pressure gradient with the corresponding forward flows, suggesting a pushing process. These suggest that the pumping at the right ventricle combines suction and pushing mechanisms.

**Fig. 6:**
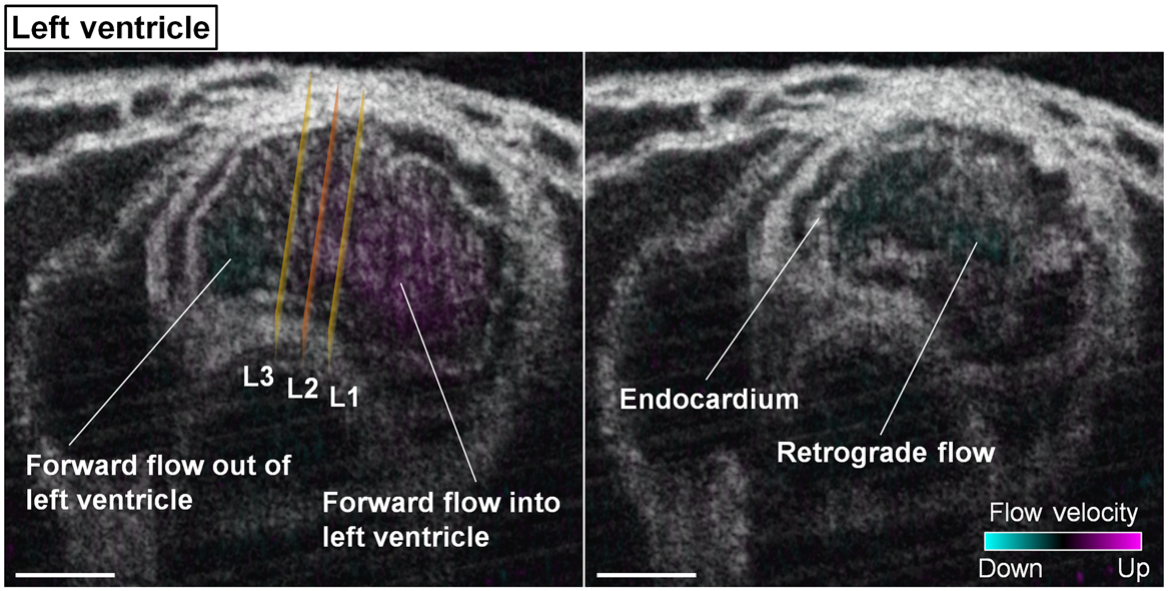
Structural and Doppler 4D-OCT imaging of the left ventricle of E9.25 embryonic heart (Video 3). The positions of the measurement planes L1, L2, and L3 are shown in the left panel. Scale bars are 100  μm (Video 3, mov, 9.96 MB [URL: https://doi.org/10.1117/1.JBO.25.8.086001.3]).

The left ventricle of the heart also revealed both forward and retrograde flows, as shown in Fig. 6 and Video 3. The forward flow was detected during about 90% of the heartbeat cycle. The retrograde flow, which lasted for about 10% of the heartbeat cycle, was detected at the last fraction of the luminal contraction phase.

**Fig. 7:**
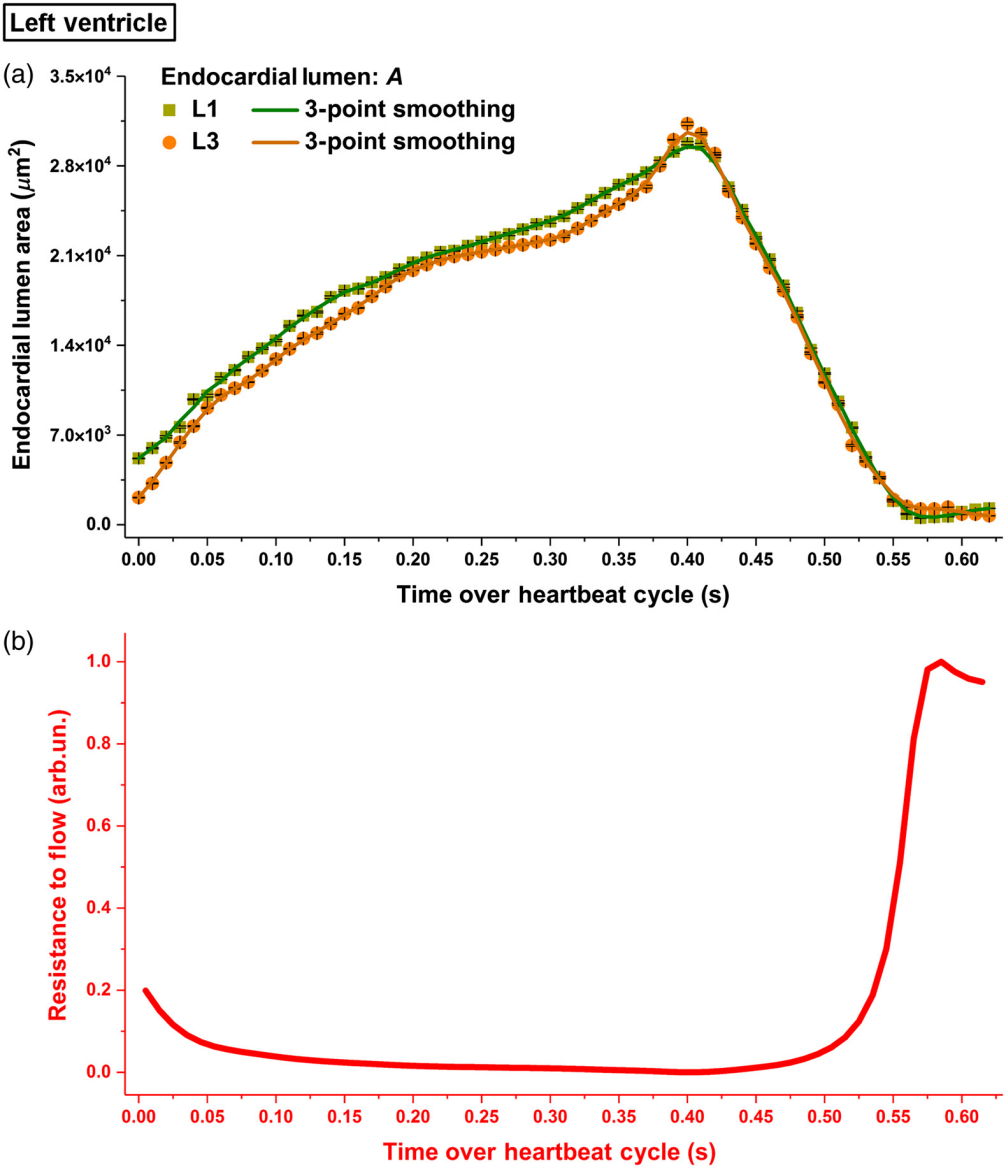
(a) Temporal profiles of the endocardial lumen area at L1 and L3 in the left ventricle of E9.25 embryonic heart and (b) the corresponding normalized resistance to flow between L1 and L3. The luminal area in (a) is plotted as mean±std. Data are from one embryo.

The temporal profiles of the endocardial lumen area and the flow resistance are shown in Fig. 7. Similar to the right ventricle, the cardiac wall activities at the left ventricle also featured a slower relaxation process in contrast to a faster contraction. Both L1 and L3 reached the most relaxed points at around the same time and had a very similar rate of luminal area change during contraction. However, over the luminal expansion, different rates were observed at L1 and L3 with L3 having a more distinguished temporal variation. Although the area at L3 was smaller than that at L1 for the majority of the cycle, it reached a higher value at the peak. The resistance to flow reached its maximum at the last phase of contraction with the luminal area close to zero.

**Fig. 8:**
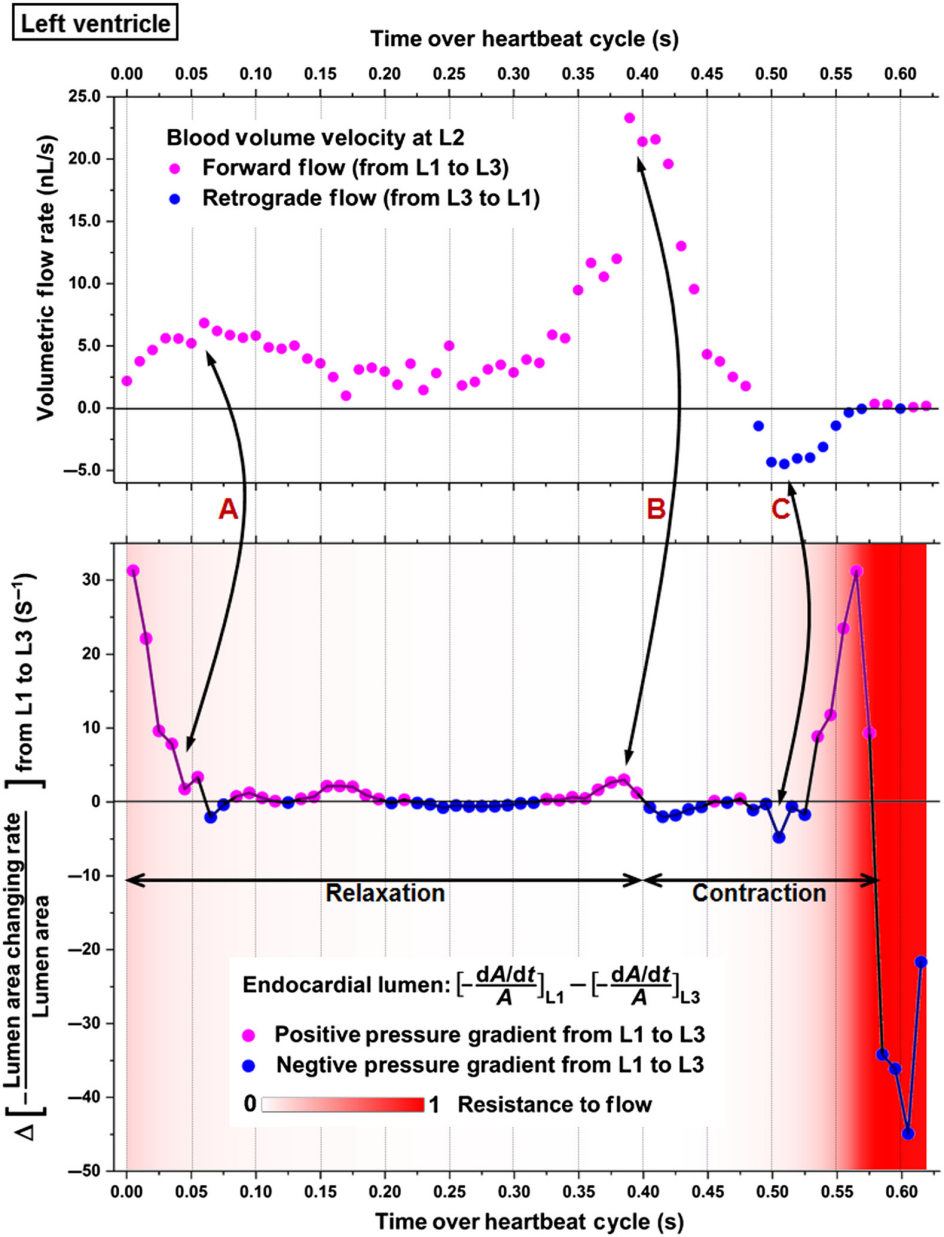
The relation between pressure gradient, resistance to flow, and volumetric flow rate in the left ventricle of E9.25 embryonic heart. Data are from one embryo.

For the left ventricle, the volumetric blood flow rate at L2 as well as the heart-wall-movement-induced pressure gradient between L1 and L3 are presented in Fig. 8. The resistance to flow is color coded and overlaid with the pressure gradient profile. The beginning of the heart wall relaxation produced positive pressure gradient from L1 to L3 with strong forward flows at L2 (double arrow A). As the lumen at L3 rapidly expanded, it generated a peak of the positive pressure gradient from L1 to L3 that coincided with the maximum of the forward flow rate (double arrow B). At the second half of the luminal contraction phase, a transient increase of the negative pressure magnitude from L1 to L3 was detected at nearly the same time as the retrograde flow reached its peak (double arrow C). Over the most contracted state, although large pressure gradients were seen between L1 and L3, the strong flow resistance lasting for at least ∼75  ms maintained the flow rate close to zero. Granger causality test was performed on the pressure gradient and volumetric flow rate at the heartbeat phase when the resistance to flow was relatively low (below 0.1). The statistical significance was detected at lag values of 1 and 2 in the direction from the pressure gradient to the flow (Table 2), suggesting that the pressure gradient induced by heart wall movements produced the local blood flow in the left ventricle. As marked in Fig. 8, the forward flows were mostly generated with a positive pressure gradient during the relaxation of left ventricle, whereas the contraction produced retrograde flows through a negative pressure gradient. This suggests that, similar to the right ventricle, the localized pumping in the left ventricle also combines suction and pushing mechanisms.

**Table 2:** Granger causality test for the left ventricle of E9.25 embryonic heart. *p≤0.05.

Causal direction	Lag=1	Lag=2	Lag=3
Pressure gradient → flow	p=0.045*	p=0.046*	p=0.125
Flow → pressure gradient	p=0.898	p=0.855	p=0.876

**Fig. 9:**
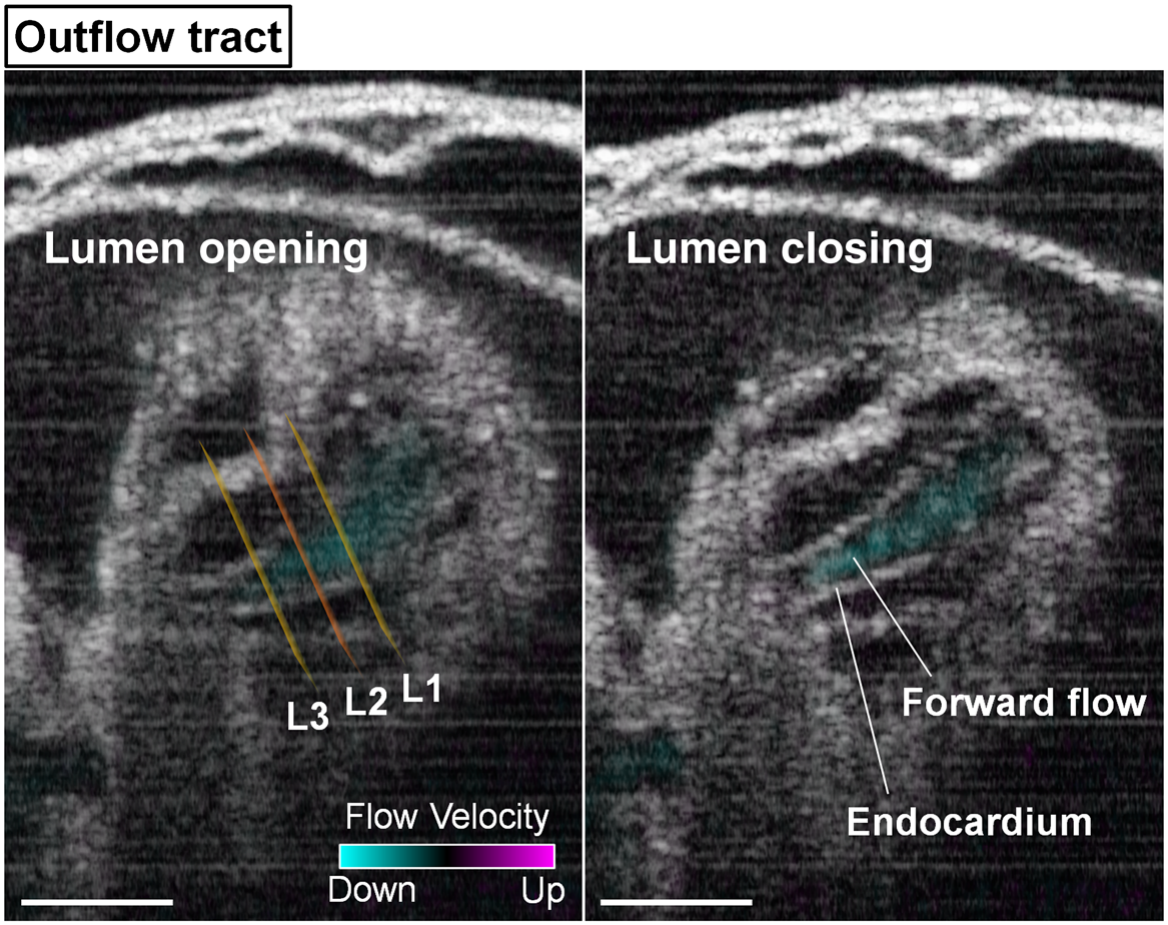
Structural and Doppler 4D-OCT imaging of the outflow tract in E9.25 embryonic heart (Video 4). The positions of the measurement planes L1, L2, and L3 are shown in the left panel. Scale bars are 100  μm (Video 4, mov, 8.64 MB [URL: https://doi.org/10.1117/1.JBO.25.8.086001.4]).

The outflow track of the heart has smaller luminal areas during opening than the ventricles. Fig. 9 and Video 4 show the combined 4D structural and hemodynamic imaging of the outflow tract, where only forward flows were observed over the heartbeat cycle.

**Fig. 10:**
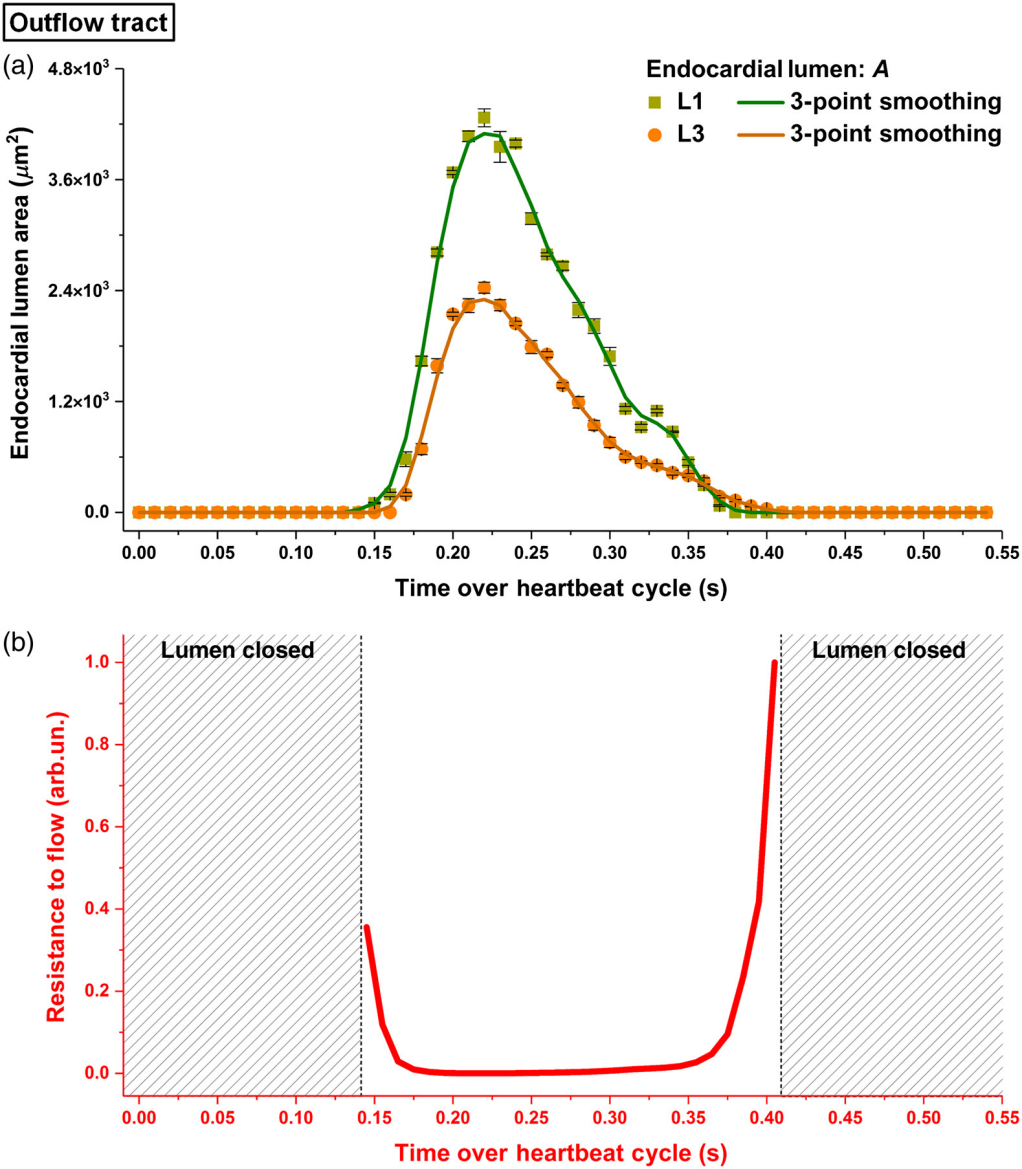
(a) Temporal profiles of the endocardial lumen area at L1 and L3 in the outflow tract of E9.25 embryonic heart and (b) the corresponding normalized resistance to flow between L1 and L3. The luminal area in (a) is plotted as mean±std. Data are from one embryo.

The temporal profiles of the endocardial lumen area in the outflow tract (Fig. 10) revealed different dynamics from the above described regions of right and left ventricles. In contrast to the slow relaxation and fast contraction in the ventricles, the outflow tract exhibited fast opening and slow closing. Another major difference detected in the outflow tract is that the time of luminal opening and closing was delayed at L3 relative to L1 by ∼20 to 30 ms. Similar to the ventricular regions, the profile for the viscous resistance in the outflow tract revealed relatively higher resistance at the beginning of luminal expansion and right before luminal closure.

**Fig. 11:**
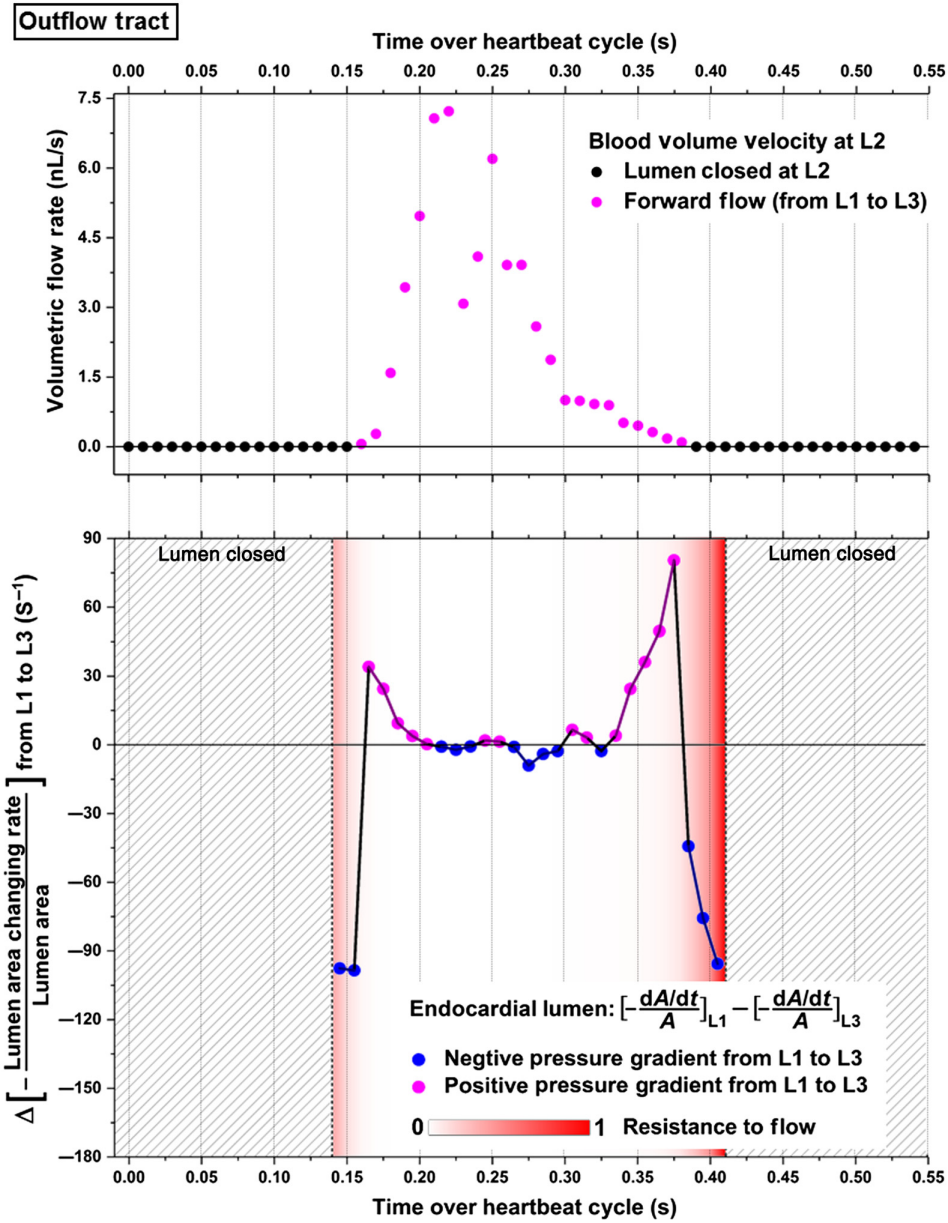
Pressure gradient, resistance to flow, and volumetric flow rate in the outflow tract of E9.25 embryonic heart. Data are from one embryo.

Figure 11 shows the pressure gradient induced by heart wall movements from L1 to L3, the flow resistance, and the volumetric flow rate at L2 of the outflow tract. No apparent relation between the pressure gradient and the blood flow were observed from these plots, except that when lumens at both L1 and L3 were just opened, a positive pressure gradient appeared at the same time as the forward flows. Moreover, the pressure gradient profile largely exhibited an overall opposite trend from the blood flow profile, suggesting the absence of a causal relation, which is confirmed by Granger causality test. No statistical significance was detected, as shown in Table 3.

**Table 3:** Granger causality test for the outflow tract of E9.25 embryonic heart.

Causal direction	Lag=1	Lag=2	Lag=3
Pressure gradient → flow	p=0.687	p=0.222	p=0.195
Flow → pressure gradient	p=0.249	p=0.552	p=0.476

The outflow tract at this embryonic stage is known to contain cardiomyocytes, which have conduction and contraction capability.^30^^,^^34^ However, these cardiomyocytes are less mature than the ones from the ventricles.^33^ In fact, they are similar to the myocytes in the initial heart tube as they are poorly coupled and contractile.^28^ On the molecular genetic level, cardiomyocytes in the outflow tract do not initiate the expression of certain genes permitting fast conduction, which, in contrast, are expressed in the early ventricles.^32^ This leaves the outflow tract slowly conducting with poor contractions until a later stage.^33^ For the mouse embryos, Chen et al. experimentally measured the conduction velocity in different heart regions from E8.5 to E10 embryonic stages. According to that study, at the E9.25 stage, the left and right ventricles have a conduction velocity of 5.5 to 10  mm/s, whereas the outflow tract has a lower conduction velocity of only 0.2 to 1.0  mm/s,^35^ as illustrated in Fig. 1. Table 4 summarizes the averaged peak blood flow speed from these three regions in E9.25 mouse embryonic hearts in our studies and the conduction velocity in corresponding regions at the same stage from Ref. 35. As one can see from Table 4, the conduction velocity in the left and right ventricles is higher than the peak blood flow speed in those regions. However, in the outflow tract, the peak blood flow speed is over five times higher than the conduction velocity. This suggests that the observed luminal dynamics in the outflow tract is a passive contractile wave with minimal contributions from localized active contraction and that the observed blood flow in the outflow tract is likely produced by the right ventricle ejection. This unique fluid-solid interaction process explains the faster luminal expansion in the outflow tract compared with the ventricles (Fig. 10). Furthermore, this agrees with our pumping assessment of the outflow tract that reveals no significant causal relation between the localized pressure gradient and the volumetric flow rate.

**Table 4:** Peak blood flow speed and conduction velocity in different regions of E9.25 embryonic heart.

Heart region	Averaged peak blood flow speed (mm/s)	Conduction velocity^35^ (mm/s)
Right ventricle	4.9	5.5 to 10
Left ventricle	3.2	5.5 to 10
Outflow tract	5.9	0.2 to 1.0

## 4 Discussion

We presented an approach for investigation of early embryonic heart pumping from the aspect of causal relation between the localized cardiodynamics and blood flow, which we applied to three distinct regions of the heart. Our pilot observations suggest that, in the ventricles where active pumping is presented, the localized heart tube functions through a combination of suction and pushing mechanisms. Such analyses could be of significance for an improved interpretation of hemodynamics in mouse mutants modeling human congenital heart defects at early embryonic stages. This method sets up a new way of studying developmental cardiac biomechanics. By building temporal profiles of critical parameters in the heart tube, evaluating their relevance, and performing causal statistical analysis, the complexity of the mechanical aspect of a beating embryonic heart can be well resolved and analyzed systematically. As biomechanical factors are increasingly recognized for their essential roles in stimulating and regulating the heart development via mechanotransduction, we hope this approach could inspire new ideas and innovative designs in imaging and measurement techniques to assess the embryonic cardiac biomechanics.

The important features of OCT in 3D imaging, deep penetration, microscale resolution, and high sampling rate, enable robust analysis of embryonic heart in the mouse model. Mice are the most popular mammalian research organism with well-established genetic engineering tools for mutagenesis, and thousands of mutant mouse lines modeling different aspects of congenital heart diseases are already available.^42^ This stimulates a critical need for methods toward investigation of cardiac development in mouse embryos structurally and functionally. The presented method can be useful for functional characterizations of the early heart dynamics, in combination with molecular genetic tools, to enable interesting studies of novel functional cardiac phenotypes in mammalian embryos.

Granger causality test is a prediction-based statistical method to assess the causal relation of time-series data and has recently found emerging applications in neuroscience and neuroimaging.^41^ In our study, we employed the Granger causality test for statistical evaluations of the localized causal relation between the dynamics of pressure gradient induced by heart wall movements and the blood flow. It is worth noting that the results from this statistical test should be interpreted in the context of the biomechanical model and biological process for understanding the localized pumping dynamics in the tubular embryonic heart. Implementation of this statistical assessment has a potential to resolve a number of controversies in cardiac developmental biology, as it is currently not clear at what developmental stage and how specific regions of the heart tube start contributing to the local and overall flow dynamics, and how the functions of different cardiac regions are integrated in the valveless heart tube. It will also be indispensable for the analysis of mutants with regional functional cardiac defects, such as the Mlc2a (atrial-specific myosin light chain 2a) knockout model,^43^ to understand the localized functional role of specific proteins in the mammalian cardiac development.

The presented approach can potentially be improved in multiple aspects. In this study, we employed OCT imaging with a B-scan rate of 100 Hz. Through postacquisition synchronization,^20^ the entire embryonic heart was reconstructed in 4D with an equivalent 100 Hz volume rate. This enabled a sufficient resolvability in time to capture the fast cardiac activities. As the pumping assessment largely relies on temporal features, a higher sampling rate is expected to strengthen the presented analysis and improve the accuracy. Segmentation of the endocardial lumen in OCT images was performed manually in this study, which is a commonly used approach and has been employed to produce the ground truths for developing automatic algorithms.^26^^,^^44^^,^^45^ Recent advancements in automatic segmentation of the *Drosophila* cardiac lumen based on convolutional neural networks^44^^,^^45^ could potentially help to reduce the time required for luminal area measurements in the early mouse heart. The measured axial resolution of OCT was lower than the theoretical value, which was likely caused by dispersion. The axial resolution could potentially be improved and optimized through advanced approaches for k-space recalibration and dispersion compensation.^46^^,^^47^ The presented method in this study can be integrated with a number of manipulation methods for embryonic heart, such as banding of the heart tube^48^ as well as optical pacing with either infrared light^49^ or optogenetics,^50^ which will bring advanced knowledge of how cardiac pumping responds to physical interventions.

The described approach requires certain assumptions for the parameters in the biomechanical model.^27^ In particular, the blood viscosity was assumed constant since it is challenging to perform spatiotemporal measurements of blood viscosity inside the embryonic heart. As a result, both the pressure gradient induced by heart wall movements and the resistance to flow were qualitatively assessed. Although the effects of these two parameters on the volumetric blood flow were evaluated integratively, the assumption of constant blood viscosity could bring errors to the analysis, which will need to be further investigated.

The method focuses on assessing the localized pumping process in the cardiac region with relatively homogeneous electrophysiological characteristics. This provides detailed delineations of the pumping dynamics within a region of ∼60  μm in length. From a larger scale, different regions of the heart work together to achieve the proper blood flows.^1^^,^^6^^,^^51^ Thus it is also important to access the dynamic coupling between different cardiac regions and to understand how the tubular heart functions as a whole. The presented approach can potentially be further developed with more measurement planes and larger distances between the planes for the pumping analysis covering the entire heart. This could eventually lead to a powerful multiscale biomechanical characterization tool to study the mammalian tubular embryonic heart.

The newly proposed method has been demonstrated in application to three distinct regions of the embryonic heart tube, revealing intriguing observations about the causal relation between localized blood flow and heart wall dynamics. Due to the limited sample size, drawing biological conclusions on cardiodynamic mechanisms is premature based on this study alone. The biological insights revealed from this pilot investigation will be further validated with a larger sample size in our future work. Nevertheless, this study demonstrated the richness of data provided by this approach and revealed a great potential for investigating the regional functional relation between blood flow and heart wall dynamics as well as studying the coupling and interplay between cardiac regions during early development, which are currently not accessible by other methods.

The embryonic heart development is continuous and rapid, with molecular, cellular, structural, and functional changes taking place within hours. To fully understand the pumping dynamics over the early cardiogenesis, 4D imaging of the mouse embryonic heart over development over the course of hours can be performed and will provide unprecedented information describing the function and mechanics of the early mammalian heart as it develops and remodels. Our previous work in long-term imaging of the mouse embryonic neural tube closure indicates the feasibility of repeated OCT imaging for over 16 h with the current experimental set up.^37^ Future work will focus on longitudinal imaging and pumping assessment of the mouse embryonic heart over E8.5–E9.5, which will be promising to produce exciting insights into the biomechanics of mammalian cardiogenesis.

## 5 Conclusion

We presented an approach for functional assessment of the localized cardiac pumping dynamics in the mouse tubular embryonic heart based on combined 4D structural and hemodynamic imaging with Doppler OCT. The method allows for characterizing the temporal connection and causal relation between the volumetric blood flow and the local pressure gradient induced by heart wall movements in the context of flow resistance within the beating heart tube. Our results show that this imaging-based approach is useful to analyze how the early mammalian heart works at specific regions, which can open new opportunities for understanding both normal and defected cardiac development at early embryonic stages.

## References

[1] Männer J. Wessel A. Yelbuz T. M., “How does the tubular embryonic heart work? Looking for the physical mechanism generating unidirectional blood flow in the valveless embryonic heart tube,” Dev. Dyn. 239, 1035–1046 (2010). DEDYEI 1097-0177 10.1002/dvdy.22265 20235196

[2] Patten B. M. Kramer T. C., “The initiation of contraction in the embryonic chick heart,” Am. J. Anat. 53, 349–375 (1933). 10.1002/aja.1000530302

[3] Goss C. M., “The physiology of the embryonic mammalian heart before circulation,” Am. J. Physiol.-Legacy Content 137, 146–152 (1942). 10.1152/ajplegacy.1942.137.1.146

[4] Hu N. Clark E. B., “Hemodynamics of the stage 12 to stage 29 chick embryo,” Circ. Res. 65, 1665–1670 (1989). 10.1161/01.RES.65.6.1665 2582595

[r5] Hu N. et al., “Diastolic filling characteristics in the stage 12 to 27 chick embryo ventricle,” Pediatr. Res. 29, 334–337 (1991). PEREBL 0031-3998 10.1203/00006450-199104000-00002 1852525

[r6] Forouhar A. S. et al., “The embryonic vertebrate heart tube is a dynamic suction pump,” Science 312, 751–753 (2006). SCIEAS 0036-8075 10.1126/science.1123775 16675702

[7] Butcher J. T. et al., “Transitions in early embryonic atrioventricular valvular function correspond with changes in cushion biomechanics that are predictable by tissue composition,” Circ. Res. 100, 1503–1511 (2007). 17478728 10.1161/CIRCRESAHA.107.148684

[r8] McQuinn T. C. et al., “High-frequency ultrasonographic imaging of avian cardiovascular development,” Dev. Dyn. 236, 3503–3513 (2007). DEDYEI 1097-0177 10.1002/dvdy.21357 17948299

[9] Srinivasan S. et al., “Noninvasive, in utero imaging of mouse embryonic heart development with 40-MHz echocardiography,” Circulation 98, 912–918 (1998). CIRCAZ 0009-7322 10.1161/01.CIR.98.9.912 9738647

[10] Michael L. et al., “Four-dimensional cardiac imaging in living embryos via postacquisition synchronization of nongated slice sequences,” J. Biomed. Opt. 10, 054001 (2005). JBOPFO 1083-3668 10.1117/1.2061567 16292961

[11] Liebling M. et al., “Rapid three-dimensional imaging and analysis of the beating embryonic heart reveals functional changes during development,” Dev. Dyn. 235, 2940–2948 (2006). DEDYEI 1097-0177 10.1002/dvdy.20926 16921497

[12] Voleti V. et al., “Real-time volumetric microscopy of in vivo dynamics and large-scale samples with SCAPE 2.0,” Nat. Methods 16, 1054–1062 (2019).1548-7091 10.1038/s41592-019-0579-4 31562489 PMC6885017

[13] Huang D. et al., “Optical coherence tomography,” Science 254, 1178–1181 (1991). SCIEAS 0036-8075 10.1126/science.1957169 1957169 PMC4638169

[14] Wang S. Larina I. V. Larin K. V., “Label-free optical imaging in developmental biology [Invited],” Biomed. Opt. Express 11, 2017–2040 (2020). BOEICL 2156-7085 10.1364/BOE.381359 32341864 PMC7173889

[15] Midgett M. Goenezen S. Rugonyi S., “Blood flow dynamics reflect degree of outflow tract banding in Hamburger–Hamilton stage 18 chicken embryos,” J. R. Soc. Interface 11, 20140643 (2014).1742-5689 10.1098/rsif.2014.0643 25165602 PMC4191090

[r16] Lopez A. L. et al., “Live four-dimensional optical coherence tomography reveals embryonic cardiac phenotype in mouse mutant,” J. Biomed. Opt. 20, 090501 (2015). JBOPFO 1083-3668 10.1117/1.JBO.20.9.090501 26385422 PMC4681392

[r17] Alex A. et al., “A Circadian clock gene, cry, affects heart morphogenesis and function in drosophila as revealed by optical coherence microscopy,” PLoS One 10, e0137236 (2015). POLNCL 1932-6203 10.1371/journal.pone.0137236 26348211 PMC4565115

[18] Ford S. M. et al., “Increased regurgitant flow causes endocardial cushion defects in an avian embryonic model of congenital heart disease,” Congenit. Heart Dis. 12, 322–331 (2017). 10.1111/chd.12443 28211263 PMC5467887

[19] Grishina O. A. Wang S. Larina I. V., “Speckle variance optical coherence tomography of blood flow in the beating mouse embryonic heart,” J. Biophotonics 10, 735–743 (2017). 10.1002/jbio.201600293 28417585 PMC5565627

[20] Wang S. et al., “Four-dimensional live imaging of hemodynamics in mammalian embryonic heart with Doppler optical coherence tomography,” J. Biophotonics 9, 837–847 (2016). 10.1002/jbio.201500314 26996292 PMC5152918

[21] Wang S. et al., “Direct four-dimensional structural and functional imaging of cardiovascular dynamics in mouse embryos with 1.5 MHz optical coherence tomography,” Opt. Lett. 40, 4791–4794 (2015). OPLEDP 0146-9592 10.1364/OL.40.004791 26469621 PMC4849121

[22] Haack T. Abdelilah-Seyfried S., “The force within: endocardial development, mechanotransduction and signalling during cardiac morphogenesis,” Development 143, 373–386 (2016). 10.1242/dev.131425 26839341

[23] Lindsey S. E. Butcher J. T. Yalcin H. C., “Mechanical regulation of cardiac development,” Front. Physiol. 5 (2014). FROPBK 0301-536X 10.3389/fphys.2014.00318 PMC4140306 25191277

[24] Liu A. et al., “Biomechanics of the chick embryonic heart outflow tract at HH18 using 4D optical coherence tomography imaging and computational modeling,” PLoS One 7, e40869 (2012). POLNCL 1932-6203 10.1371/journal.pone.0040869 22844414 PMC3402486

[r25] Sandra R. et al., “Changes in wall motion and blood flow in the outflow tract of chick embryonic hearts observed with optical coherence tomography after outflow tract banding and vitelline-vein ligation,” Phys. Med. Biol. 53, 5077 (2008). PHMBA7 0031-9155 10.1088/0031-9155/53/18/015 18723935

[26] Peterson L. M. et al., “4D shear stress maps of the developing heart using Doppler optical coherence tomography,” Biomed. Opt. Express 3, 3022–3032 (2012). BOEICL 2156-7085 10.1364/BOE.3.003022 23162737 PMC3493225

[27] Bulk A. et al., “Mechanisms influencing retrograde flow in the atrioventricular canal during early embryonic cardiogenesis,” J. Biomech. 49, 3162–3167 (2016). 10.1016/j.jbiomech.2016.07.028 27511597

[28] Moorman A. F. M. Christoffels V. M., “Cardiac chamber formation: development, genes, and evolution,” Physiol. Rev. 83, 1223–1267 (2003). PHREA7 0031-9333 10.1152/physrev.00006.2003 14506305

[r29] Goenezen S. Rennie M. Y. Rugonyi S., “Biomechanics of early cardiac development,” Biomech. Model. Mechanobiol. 11, 1187–1204 (2012). BMMICD 1617-7959 10.1007/s10237-012-0414-7 22760547 PMC3475730

[30] van Weerd J. H. Christoffels V. M., “The formation and function of the cardiac conduction system,” Development 143, 197–210 (2016). 10.1242/dev.124883 26786210

[31] Boucek R. J. Murphy W. P. Paff G. H., “Electrical and mechanical properties of chick embryo heart chambers,” Circ. Res. 7, 787–793 (1959). 13803174 10.1161/01.res.7.5.787

[r32] de Jong F. et al., “Persisting zones of slow impulse conduction in developing chicken hearts,” Circ. Res. 71, 240–250 (1992). 10.1161/01.RES.71.2.240 1628384

[33] Boukens B. J. D. et al., “Developmental basis for electrophysiological heterogeneity in the ventricular and outflow tract myocardium as a substrate for life-threatening ventricular arrhythmias,” Circ. Res. 104, 19–31 (2009). 19118284 10.1161/CIRCRESAHA.108.188698

[34] Christoffels V. M. Moorman A. F. M., “Development of the cardiac conduction system,” Circ.: Arrhythmia Electrophysiol. 2, 195–207 (2009). 10.1161/CIRCEP.108.829341 19808465

[35] Chen F. et al., “Atrioventricular conduction and arrhythmias at the initiation of beating in embryonic mouse hearts,” Dev. Dyn. 239, 1941–1949 (2010). DEDYEI 1097-0177 10.1002/dvdy.22319 20549739 PMC2908293

[36] Lopez A. L. et al., “Live confocal microscopy of the developing mouse embryonic yolk sac vasculature,” in Vascular Morphogenesis: Methods and Protocols, Ribatti D., Ed., pp. 163–172, Springer, New York (2015). 10.1007/978-1-4939-1462-3_9 25468603

[37] Wang S. et al., “Dynamic imaging and quantitative analysis of cranial neural tube closure in the mouse embryo using optical coherence tomography,” Biomed. Opt. Express 8, 407–419 (2016). BOEICL 2156-7085 10.1364/BOE.8.000407 28101427 PMC5231309

[38] de Soysa T. Y. et al., “Single-cell analysis of cardiogenesis reveals basis for organ-level developmental defects,” Nature 572, 120–124 (2019). 10.1038/s41586-019-1414-x 31341279 PMC6719697

[39] Yang V. X. D. et al., “High speed, wide velocity dynamic range Doppler optical coherence tomography (Part I): system design, signal processing, and performance,” Opt. Express 11, 794–809 (2003). OPEXFF 1094-4087 10.1364/OE.11.000794 19461792

[40] Jenkins M. W. et al., “Measuring hemodynamics in the developing heart tube with four-dimensional gated Doppler optical coherence tomography,” J. Biomed. Opt. 15, 066022 (2010). JBOPFO 1083-3668 10.1117/1.3509382 21198196 PMC3017576

[41] Seth A. K. Barrett A. B. Barnett L., “Granger causality analysis in neuroscience and neuroimaging,” J. Neurosci. 35, 3293–3297 (2015). JNRSDS 0270-6474 10.1523/JNEUROSCI.4399-14.2015 25716830 PMC4339347

[42] Kamp A. et al., “Genome-wide identification of mouse congenital heart disease loci,” Hum. Mol. Genet. 19, 3105–3113 (2010). HMGEE5 0964-6906 10.1093/hmg/ddq211 20511334 PMC2908466

[43] Huang C. et al., “Embryonic atrial function is essential for mouse embryogenesis, cardiac morphogenesis and angiogenesis,” Development 130, 6111–6119 (2003). 10.1242/dev.00831 14573518

[44] Duan L. et al., “Segmentation of Drosophila heart in optical coherence microscopy images using convolutional neural networks,” J. Biophotonics 11, e201800146 (2018). 10.1002/jbio.201800146 29992766 PMC6289629

[45] Dong Z. et al., “FlyNet 2.0: drosophila heart 3D (2D + time) segmentation in optical coherence microscopy images using a convolutional long short-term memory neural network,” Biomed. Opt. Express 11, 1568–1579 (2020). BOEICL 2156-7085 10.1364/BOE.385968 32206429 PMC7075608

[46] Singh K. Sharma G. Tearney G. J., “Estimation and compensation of dispersion for a high-resolution optical coherence tomography system,” J. Opt. 20, 025301 (2018). 10.1088/2040-8986/aaa2c0

[47] Xavier A. et al., “Simple and robust calibration procedure for k-linearization and dispersion compensation in optical coherence tomography,” J. Biomed. Opt. 24, 1–11 (2019). JBOPFO 1083-3668 10.1117/1.JBO.24.5.056001 PMC6992960 31087833

[48] Stovall S. et al., “Changes in dynamic embryonic heart wall motion in response to outflow tract banding measured using video densitometry,” J. Biomed. Opt. 21, 116003 (2016). JBOPFO 1083-3668 10.1117/1.JBO.21.11.116003 27812694 PMC5795889

[49] Jenkins M. W. et al., “Optical pacing of the embryonic heart,” Nat. Photonics 4, 623–626 (2010). NPAHBY 1749-4885 10.1038/nphoton.2010.166 21423854 PMC3059323

[50] Alex A. et al., “Optogenetic pacing in *Drosophila melanogaster*,” Sci. Adv. 1, e1500639 (2015). STAMCV 1468-6996 10.1126/sciadv.1500639 26601299 PMC4646813

[51] Bark D. L. et al., “Valveless pumping mechanics of the embryonic heart during cardiac looping: pressure and flow through micro-PIV,” J. Biomech. 50, 50–55 (2017). JBMCB5 0021-9290 10.1016/j.jbiomech.2016.11.036 27887729

